# An Inequality of Meromorphic Functions and Its Application

**DOI:** 10.1155/2014/242851

**Published:** 2014-03-06

**Authors:** Zhaojun Wu, Yuxian Chen, Zuxing Xuan

**Affiliations:** ^1^School of Mathematics and Statistics, Hubei University of Science and Technology, Xianning 437100, China; ^2^School of Mathematics and Computer Science, Xinyu University, Xinyu 338004, China; ^3^Beijing Key Laboratory of Information Service Engineering, Department of General Education, Beijing Union University, No. 97 Bei Si Huan Dong Road, Chaoyang District, Beijing 100101, China

## Abstract

By applying Ahlfors theory of covering surface, we establish a fundamental inequality of meromorphic function dealing with multiple values in an angular domain. As an application, we prove the existence of some new singular directions for a meromorphic function *f*, namely a Bloch direction and a pseudo-T direction for *f*.

## 1. Introduction

In this paper, meromorphic function always means a function meromorphic in the whole complex plane. Given a meromorphic function *f*(*z*), the theory of value distribution of *f*(*z*) developed in the two ways: one is the module distribution and the other is angular distribution. For the module distribution of a meromorphic function, there are three main theorems, that is, the Picard theorem, the Borel theorem, and the Nevanlinna second fundamental theorem. The fundamental concept in the angular distribution is singular direction. Singular direction is a concept of localizing value distribution in *ℂ* onto a sector *S* containing a single ray *J* : arg*z* = *θ* emanating from the origin say. A Julia direction and a Borel direction are refinements of the Picard theorem and the Borel theorem, respectively. Corresponding to the Nevanlinna second fundamental theorem, a new singular direction, called T direction, was recently introduced in Zheng [1]. When multiple values were considered, Yang [2] proved the following theorems related to the module distribution of meromorphic function. In order to introduce the main results of Yang, we give some notations (see [2]) as the following.

Let *f*(*z*) denote a nonconstant meromorphic function, *a* ∈ *ℂ* an arbitrary complex number, and *k* a positive integer. We use *n* ^*k*)^(*r*, 1/(*f* − *a*)) or *n* ^*k*)^(*r*, *a*) to denote the zeros of *f*(*z*) − *a* in |*z* | ≤*r*, whose multiplicities are no greater than *k*, counted according to their multiplicities. Likewise, we use *n* ^(*k*^(*r*, 1/(*f* − *a*)) or *n* ^(*k*^(*r*, *a*) to denote those zeros in |*z* | ≤*r*, whose multiplicities are greater than *k*, counted according to their multiplicities. The corresponding counting functions are denoted by *N* ^*k*)^(*r*, 1/(*f* − *a*)) or *N* ^*k*)^(*r*, *a*) and *N* ^(*k*^(*r*, 1/(*f* − *a*)) or *N* ^(*k*^(*r*, *a*). Let *f*(*z*) be a meromorphic function with order *ρ*  (0 < *ρ* < +*∞*), *a* be an arbitrary number, and *k* be a positive integer. If

(1) $$\begin{array}{c} \underset{r\to \infty }{\mathrm{limsup}}\frac{\log n^{k)}\left(r,a\right)}{\log r}<\rho , \end{array}$$

then *a* is called a pseudo-Borel exceptional value of *f*(*z*) of order *k*.

In [2], Yang has proved the following theorems.

**Theorem 1 A**

Let *f*(*z*) be a meromorphic function with order *ρ*  (0 < *ρ* < +*∞*) and let *k*_*j*_  (*j* = 1,2,…, *q*) be *q* positive integers. If *f*(*z*) has *q* distinct pseudo-Borel exceptional values *a*_*j*_ of order *k*_*j*_(*j* = 1,2,…, *q*), then

(2) $$\begin{array}{c} \sum_{j=1}^{q}\left(1-\frac{1}{k_{j}+1}\right)\leq 2. \end{array}$$

**Theorem 1 B**

Let *f*(*z*) be a nonconstant meromorphic, *a*_*j*_ ∈ *ℂ*_*∞*_  (*j* = 1,2,…, *q*) be *q*(≥3) distinct complex numbers, and *k*_*j*_  (*j* = 1,2,…, *q*) be *q* positive integers. Then

(3) (∑j=1q(1−1kj+1)−2)T(r,f)  <∑j=1qkjkj+1Nkj)(r,1f−aj)+S(r,f),

where *S*(*r*, *f*) is the Nevanlinna error term.

In this paper, we will research the singular directions corresponding to Theorems A and B.

## 2. A Theorem on Covering Surface

In this section, we will give a theorem on covering surface. We firstly introduce the following notations (see Tsuji [3]).

In this paper, the Riemann sphere of diameter 1 is denoted by *K*. Let *F* be a finite covering surface of *F*_0_, consisting of a finite number of sheets, and be bounded by a finite number of analytic Jordan curves {Λ_*j*_} (some of which may reduce to single points), and the spherical distance between any two circular curves Λ_*i*_ and Λ_*j*_ is *d*(Λ_*i*_, Λ_*j*_) ≥*δ* ∈ (0,1/2). The part of the boundary of *F*, which does not lie above the boundary of *F*_0_, is called the relative boundary of *F* and denote its spherical length by *L*. Let *D* be a domain on *F*_0_, whose boundary consists a finite number of points or analytic closed Jordan curves, and let *F*(*D*) be the part of *F*, which lies above *D*. We denote the spherical area of *F*, *F*(*D*), and *F*_0_ by |*F*|, |*F*(*D*)| and |*F*_0_|, respectively. We put

(4) $$\begin{array}{c} S=\frac{\left|F\right|}{\left|F_{0}\right|},\;\;S\left(D\right)=\frac{\left|F\left(D\right)\right|}{\left|D\right|}. \end{array}$$

Under the above notation, we have the following Ahlfors covering Theorem.

**Lemma 1 (see Tsuji [3])**

For any finite covering surface *F* of *F*_0_, one has

(5) $$\begin{array}{c} \left|S-S\left(D\right)\right|<h\frac{L}{\left|D\right|}, \end{array}$$

where *h* > 0 is a constant which depends on *F*_0_ only.

Recently, Sun [4] has proved a precise version of Lemma 1 and proved that *h* = 2*π*/*δ*, where 0 < *δ* < 1/2 is a constant.

**Lemma 2 (see Sun [5])**

Let *F* be a simply connected finite covering surface of the unite sphere *K*, and let {*D*_*v*_} be *q*(>2) disjoint spherical disks on *K*, where the spherical distance of any pair of {*D*_*v*_} is at least *δ*. Let *n*_*v*_ be the number of simply connected islands (see Tsuji [3, Page 252]) in *F*(*D*_*v*_)); then

(6) $$\begin{array}{c} \sum_{v=1}^{q}n_{v}\geq \left(q-2\right)S-\frac{C}{\delta^{3}}L, \end{array}$$

where *L* is the length of the relative boundary of *F* and *C* is a constant.

**Theorem 3**

Let *F* be a simply connected finite covering surface of the unite sphere *K*, and let *l*_*v*_  (*v* = 1,2,…, *q*) be *q* positive integers. Let *D*_*v*_  (*v* = 1,2,…, *q*) be *q*(>2) disjoint spherical disks with radius *δ*/3 on *K* and without a pair of  {*D*_*v*_} such that their spherical distance is less than *δ* and let *n*_*v*_^*l*_*v*_)^ be the number of simply connected islands in *F*(*D*_*v*_), which consisted of no more than *l*_*v*_ sheets; then

(7) ∑v=1qlvlv+1nvlv)≥(∑j=1q(1−1lv+1)−2)S −C+9qhδ3L,

where *L* is the length of the relative boundary of *F*.

**Proof**

It is easy to verify that

(8) $$\begin{array}{c} n_{v}=n_{v}^{l_{v})}+n_{v}^{(l_{v}},\;\;S\left(D_{v}\right)\geq n_{v}^{l_{v})}+\left(l+1\right)n_{v}^{(l_{v}}, \end{array}$$

where *n*_*v*_^(*l*_*v*_^ is the number of simply connected islands in *F*(*D*_*v*_), which consist of no less than *l*_*v*_ + 1 sheets. Hence,

(9) $$\begin{array}{c} S\left(D_{v}\right)\geq \left(l_{v}+1\right)\left(n_{v}^{l_{v})}+n_{v}^{(l_{v}}\right)-l_{v}n_{v}^{l_{v})}=\left(l_{v}+1\right)n_{v}-l_{v}n_{v}^{l_{v})}. \end{array}$$

Since the spherical area of *D*_*v*_ is |*D*_*v*_ | ≥*δ* ^2^/9, it follows from Lemma 1 that

(10) $$\begin{array}{c} S+\frac{9h}{\delta^{2}}L>S\left(D_{v}\right)\geq \left(l_{v}+1\right)n_{v}-l_{v}n_{v}^{l_{v})}. \end{array}$$

Note that 1/(*l*_*v*_ + 1) < 1 and 0 < *δ* < 1/2; we can get

(11) $$\begin{array}{c} n_{v}\leq \frac{l_{v}}{l_{v}+1}n_{v}^{l_{v})}+\frac{1}{l_{v}+1}S+\frac{9h}{\delta^{3}}L. \end{array}$$

Adding two sides of the above expression from 1 to *q*, we have

(12) $$\begin{array}{c} \sum_{v=1}^{q}n_{v}\leq \sum_{v=1}^{q}\frac{l_{v}}{l_{v}+1}n_{v}^{l_{v})}+\sum_{v=1}^{q}\frac{1}{l_{v}+1}S+\frac{9qh}{\delta^{3}}L. \end{array}$$

Combining Lemma 2 and the above expression, Theorem 3 follows.

## 3. A Fundamental Inequality of Meromorphic Functions in an Angular Domain

The Ahlfors-Shimizu characteristic is important in this paper. Let us recall its definition. Suppose that *E* is a nonempty subset of *ℂ*; we denote

(13) $$\begin{array}{c} S\left(r,E,f\right)=\frac{1}{\pi }\int \int_{E}{\left(\frac{\left|f^{\prime }\left(z\right)\right|}{1+{\left|f\left(z\right)\right|}^{2}}\right)}^{2}dw,\\ T_{0}\left(r,E,f\right)=\int_{0}^{r}\frac{S\left(t,E,f\right)}{t}dt. \end{array}$$

When *E* = *ℂ*, we write *T*(*r*, *ℂ*, *f*) by *T*_0_(*r*, *f*). Then from Theorem 1.4 in [6], we have

(14) $$\begin{array}{c} \left|T\left(r,f\right)-\log^{+}\left|f\left(0\right)\right|-T_{0}\left(r,f\right)\right|\leq \frac{1}{2}\log2. \end{array}$$

And the difference *T*(*r*, *f*) − *T*_0_(*r*, *f*) is a bounded function of *r*, so that both the characteristic function *T*_0_(*r*, *f*) and *T*(*r*, *f*) are interchangeable. Denote the following angular domain by

(15) $$\begin{array}{c} \Omega \left(\theta ,ɛ\right)=\left\{z\in \mathbb{C},\left|\arg z-\theta \right|<ɛ\right\}. \end{array}$$

When *E* is a sector {*z* ∈ *ℂ*, |*z* | <*r*}∩*Ω*(*θ*, *ɛ*), we denote *S*(*E*, *f*) = *S*(*r*, *Ω*(*θ*, *ɛ*), *f*) and

(16) $$\begin{array}{c} T\left(r,\Omega \left(\theta ,ɛ\right),f\right)=\int_{0}^{r}\frac{S\left(t,\Omega \left(\theta ,ɛ\right),f\right)}{t}dt. \end{array}$$

For any *a* ∈ *ℂ*_*∞*_ and *a* ≠ *∞*, let *n*(*r*, *θ*, *ɛ*, *a*) be the number of zeros, counted according to their multiplicities, of *f*(*z*) − *a* in the sector {*z* ∈ *ℂ*, |*z* | <*r*}∩*Ω*(*θ*, *ɛ*), and let *n* ^*l*)^(*r*, *θ*, *ɛ*, *a*) be the number of zeros with multiplicities ≤*l*, of *f*(*z*) − *a* in the sector {*z* ∈ *ℂ*, |*z* | <*r*}∩*Ω*(*θ*, *ɛ*), where *l* is any positive integer. Similarly, note the number of poles of *f* by *n*(*r*, *θ*, *ɛ*, *∞*) and *n* ^*l*)^(*r*, *θ*, *ɛ*, *∞*). Denote

(17) N(r,θ,ɛ,a)=∫0rn(t,θ,ɛ,a)−n(0,θ,ɛ,a)tdt +n(0,θ,ɛ,a)log⁡⁡r;Nl)(r,θ,ɛ,a)=∫0rnl)(t,θ,ɛ,a)−nl)(0,θ,ɛ,a)tdt +nl)(0,θ,ɛ,a)log⁡⁡r.

In addition, we also need the notations (see [7])

(18) L(r,ψ1,ψ2)=∫ψ1ψ2|f′(reiψ)|(1+|f(reiψ)|2)r dψ,L(r,ψ)=∫1r|f′(teiψ)|(1+|f(teiψ)|2)dt.

In this section, we will establish a fundamental inequality for meromorphic functions in an angular domain. Firstly, we give the following lemma.

**Lemma 4**

Suppose that *f*(*z*) is a meromorphic function and *l*_*v*_  (*v* = 1,2,…, *q*) be *q* positive integers, and {*a*_*v*_} are *q*(>2) distinct points on *K* and without a pair of {*a*_*v*_} such that their spherical distance is less than *δ* + 2*δ*/3. *n*_*v*_^*l*_*v*_)^ be the number of zeros of *f*(*z*) − *a*_*v*_, which are consisted of not more than *l*_*v*_ multiplicities, then

(19) $$\begin{array}{c} \sum_{v=1}^{q}\left(\frac{l_{v}}{l_{v}+1}\right)n_{v}^{l_{v})}\geq \left(\sum_{j=1}^{q}\left(1-\frac{1}{l_{v}+1}\right)-2\right)S-\frac{C+9qh}{\delta^{3}}L. \end{array}$$

**Proof**

Let *D*_*v*_ be a spherical disk with the center *a*_*v*_ with radius *δ*/3 on *K*. By Theorem 3, we have

(20) $$\begin{array}{c} \sum_{v=1}^{q}\left(\frac{l_{v}}{l_{v}+1}\right)n_{v}^{l_{v})}\geq \left(\sum_{j=1}^{q}\left(1-\frac{1}{l_{v}+1}\right)-2\right)S-\frac{C+9qh}{\delta^{3}}L. \end{array}$$

Note that *n*_*v*_^*l*_*v*_)^(*D*_*v*_) ≤ *n*_*v*_^*l*_*v*_)^(*a*_*v*_), whenever *a*_*v*_ in the island of *D*_*v*_ or in the peninsula of *D*_*v*_. Therefore, Lemma 4 follows.

We are now in the position to establish the main result in this section.

**Theorem 5**

Let *f*(*z*) be a meromorphic function and *l*_*v*_  (*v* = 1,2,…, *q*)  *q* positive integers. If {*a*_*v*_} are *q* distinct points on *K*, then one has

(21) (∑j=1q(1−1lv+1)−2)S(r,Ω(θ,φ),f)  ≤∑v=1qlvlv+1nlv)(r,θ,δ,av)   +2πH2(∑j=1q(1−1/(lv+1))−2)(δ−φ)log⁡⁡r   +(∑j=1q(1−1lv+1)−2)S(1,Ω(θ,φ),f)   +HL(1,θ−δ,θ+δ)+HL(r,θ−δ,θ+δ),

(22) (∑j=1q(1−1lv+1)−2)T(r,Ω(θ,φ),f)  ≤∑v=1qlvlv+1Nlv)(r,θ,δ,av)   +2πH2(∑j=1q(1−1/(lv+1))−2)(δ−φ)log⁡2r   +(∑j=1q(1−1lv+1)−2)T(1,Ω(θ,φ),f)   +(∑j=1q(1−1lv+1)−2)S(1,Ω(θ,φ),f)log⁡⁡r   +HL(1,θ−δ,θ+δ)log⁡⁡r+χ(r,θ−δ,θ+δ)

for any *φ*, 0 < *φ* < *δ*, where *H* is a constant depending only on *a*_*v*_, *v* = 1,2,…, *q*, and *χ*(*r*, *θ* − *δ*, *θ* + *δ*) = *H*∫_1_^*r*^(*L*(*t*, *θ* − *δ*, *θ* + *δ*)/*t*)*dt*.

**Proof**

Put *D*_*r*_ = {*z* ∈ *ℂ*, 1 < |*z* | <*r*}∩*Ω*(*θ*, *φ*) and *F*_0_ = *K* − {*a*_*v*_}. Using Lemma 4, we have

(23) (∑j=1q(1−1lv+1)−2)[S(r,Ω(θ,φ),f)−S(1,Ω(θ,φ),f)]  ≤∑v=1qlvlv+1nlv)(r,θ,δ,av)+HL(r),

where *H* = (*C* + 9*qh*)/*lδ* ^3^, which depends only on *F*_0_, that is, only on *a*_*v*_, *v* = 1,2,…, *q*, and

(24) L(r)=L(r,θ−φ,θ+φ)+L(1,θ−φ,θ+φ) +L(r,θ−φ)+L(r,θ+φ)≤L(r,θ−δ,θ+δ)+L(1,θ−δ,θ+δ) +L(r,θ−φ)+L(r,θ+φ).

Hence

(25) (∑j=1q(1−1lv+1)−2)  ×[S(r,Ω(θ,φ),f)−S(1,Ω(θ,φ),f)]  −∑v=1qlvlv+1nlv)(r,θ,δ,av)−HL(r,θ−δ,θ+δ)  −HL(1,θ−δ,θ+δ) ≤H[L(r,θ−φ)+L(r,θ+φ)].

Denote the left expression of (25) by *A*(*r*, *φ*); thus

(26) d(A(r,φ))dφ  =(∑j=1q(1−1lv+1)−2)   ×d[S(r,Ω(θ,φ),f)−S(1,Ω(θ,φ),f)]dφ.

We claim the fact that

(27) [L(r,θ−φ)+L(r,θ+φ)]2≤2π(∑j=1q(1−1/(lv+1))−2) ×d(A(r,φ))dφlog⁡⁡r.

In fact, it follows from the definition of *L*(*r*, *ψ*) and Schwarz's inequality that

(28) [L(r,θ−φ)+L(r,θ+φ)]2 ≤2[(∫1r|f′(tei(θ−φ))|(1+|f(tei(θ−φ))|2)dt)2+(∫1r|f′(tei(θ+φ))|(1+|f(tei(θ+φ))|2)dt)2] ≤2πd[S(r,Ω(θ,φ),f)−S(1,Ω(θ,φ),f)]dφlog⁡⁡r =2π(∑j=1q(1−1/(lv+1))−2)d(A(r,φ))dφlog⁡⁡r.

Noting *A*(*r*, *φ*) is an increasing function of *φ*, we see that then there exists a *δ*_0_ > 0, such that *A*(*r*, *φ*) > 0, when *φ* > *δ*_0_, and *A*(*r*, *φ*) ≤ 0, when *φ* ≤ *δ*_0_. For *φ* > *δ*_0_, by (25) and (27),

(29) [A(r,φ)]2≤H2[L(r,θ−φ)+L(r,θ+φ)]2≤2πH2(∑j=1q(1−1lv+1)−2)log⁡⁡rd(A(r,φ))dφ;

that is,

(30) $$\begin{array}{c} d\varphi \leq \frac{2\pi H^{2}}{\left(\sum_{j=1}^{q}\left(1-1/\left(l_{v}+1\right)\right)-2\right)}\log r\frac{d\left(A\left(r,\varphi \right)\right)}{{\left[A\left(r,\varphi \right)\right]}^{2}}. \end{array}$$

Integrating each side of the inequality leads to

(31) $$\begin{array}{c} \delta -\varphi =\int_{\varphi }^{\delta }d\varphi \leq \frac{2\pi H^{2}}{\left(\sum_{j=1}^{q}\left(1-\frac{1}{l_{v}+1}\right)-2\right)A\left(r,\varphi \right)}\log r. \end{array}$$

Thus

(32) $$\begin{array}{c} A\left(r,\varphi \right)\leq \frac{2\pi H^{2}}{\left(\sum_{j=1}^{q}\left(1-1/\left(l_{v}+1\right)\right)-2\right)\left(\delta -\tau \right)}\log r. \end{array}$$

On the case of *φ* ≤ *δ*_0_, the above inequality is obviously valid because of *A*(*r*, *φ*) ≤ 0. Replacing *A*(*r*, *φ*) in the above inequality with its explicit expression, we see that (21) is established. Therefore

(33) (∑j=1q(1−1lv+1)−2)T(r,Ω(θ,φ),f) ≤∑v=1qlvlv+1Nlv)(r,θ,δ,av)  +πH2(∑j=1q(1−1/(lv+1))−2)(δ−φ)log⁡2r    +(∑j=1q(1−1lv+1)−2)T(1,Ω(θ,φ),f)  +(∑j=1q(1−1lv+1)−2)S(1,Ω(θ,φ),f)log⁡⁡r  +HL(1,θ−δ,θ+δ)log⁡⁡r+χ(r,θ−δ,θ+δ),

where *χ*(*r*, *θ* − *δ*, *θ* + *δ*) = *H*∫_1_^*r*^(*L*(*t*, *θ* − *δ*, *θ* + *δ*)/*t*)*dt*.

**Lemma 6 (Zhang [7])**

Under the condition of Theorem 5, one has

(34) χ(r,θ−δ,θ+δ)=H∫1rL(t,θ−δ,θ+δ)tdt≤H2δπS(r,Ω(θ,δ),f)log⁡⁡r

or

(35) χ(r,θ−δ,θ+δ)≤H2δπT(r,Ω(θ,δ),f) ×log⁡⁡T(r,Ω(θ,δ),f)

with at most one exceptional set *E*_*δ*_ of *r*, where *E*_*δ*_ consists of a series of intervals and satisfies

(36) $$\begin{array}{c} \int_{E_{\delta }}\frac{1}{r\log r}dr\leq \frac{1}{\log T\left(r,\Omega \left(\theta ,,\delta \right),f\right)}<\infty . \end{array}$$

In particular, if the order of *f*(*z*) is *ρ*  (0 < *ρ* < +*∞*), then

(37) $$\begin{array}{c} \chi \left(r,\theta -\delta ,\theta +\delta \right)\leq O\left(r^{3\rho /4}\right). \end{array}$$

From Theorem 3 and Lemma 6, we can write the result in Theorem 3 as

(38) (∑j=1q(1−1lv+1)−2)T(r,Ω(θ,φ),f)  ≤∑v=1qlvlv+1Nlv)(r,θ,δ,av)   +O(log⁡2r)+χ(r,θ−δ,θ+δ).

If the order of *f*(*z*) is *ρ*  (0 < *ρ* < +*∞*), then the inequality will be

(39) (∑j=1q(1−1lv+1)−2)T(r,Ω(θ,φ),f)  ≤∑v=1qlvlv+1Nlv)(r,θ,δ,av)+O(r3ρ/4).

## 4. Bloch Direction of Meromorphic Functions

In this section, we will research the singular direction corresponding to Theorem A. Suppose that *f*(*z*) is a meromorphic function of infinite order. Then, there is a real function *ρ*(*r*) called an Hiong's proximate order (see [8]) of *f*(*z*), which has the following properties. (i) *ρ*(*r*) is continuous and nondecreasing for *r* ≥ *r*_0_  (*r*_0_ > 0) and tends to +*∞* as *r* → +*∞*. (ii) The function *U*(*r*) = *r* ^*ρ*(*r*)^(*r* ≥ *r*_0_) satisfies the condition

(40) lim⁡r→+∞log⁡⁡U(R)log⁡⁡U(r)=1,  R=r+rlog⁡⁡U(r);limsup⁡r→+∞log⁡⁡T(r,f)log⁡⁡U(r)=1.

For a meromorphic function of infinite order, Zhuang Qitai (or Chuang Chitai) [9] gives the following definition of Borel direction and Bloch direction.

**Definition 7**

Let *f*(*z*) be a meromorphic function of infinite order and *ρ*(*r*) an order of *f*(*z*). A direction arg*z* = *θ* is called a Borel direction of order *ρ*(*r*) of *f*(*z*) if, no matter how small the positive number *η* is, for each value *ω*, one has

(41) $$\begin{array}{c} \underset{r\to \infty }{\mathrm{limsup}}\frac{\log n\left(r,\theta ,\eta ,\omega \right)}{\rho \left(r\right)\log r}=1, \end{array}$$

except for at most two exceptional values *ω*. A direction arg*z* = *θ* is called a Bloch direction of order *ρ*(*r*) of *f*(*z*) if, for any number *ɛ*  (0 < *ɛ* < *π*/2), any system *a*_*j*_  (*j* = 1,2,…, *q*) of distinct values and, any system *k*_*j*_  (*j* = 1,2,…, *q*) such that *k*_*j*_ is a positive integer or +*∞* and that

(42) $$\begin{array}{c} \sum_{j=1}^{q}\left(1-\frac{1}{k_{j}+1}\right)>2, \end{array}$$

there exists at least one integer *j*  (1 ≤ *j* ≤ *q*) such that

(43) $$\begin{array}{c} \underset{r\to \infty }{\mathrm{limsup}}\frac{\log n^{k_{j})}\left(r,\theta ,ɛ,a_{j}\right)}{\rho \left(r\right)\log r}=1. \end{array}$$

For the connection of Borel direction and Bloch direction of meromorphic function of infinite order, Chuang [9] has proved the following theorem.

**Theorem 4 C**

Let *f*(*z*) be a meromorphic function of infinite order and *ρ*(*r*) an order of *f*(*z*). Then every Borel direction of order *ρ*(*r*) of *f*(*z*) is a Bloch direction of order *ρ*(*r*) of *f*(*z*).

It is natural to consider whether there exists a similar result, if meromorphic function of order infinity is replaced with meromorphic function of order *ρ*  (0 < *ρ* < +*∞*). In this section we extend the above theorem to meromorphic function of order *ρ*  (0 < *ρ* < +*∞*).

**Definition 8**

Let *f*(*z*) be a meromorphic function of order *ρ*  (0 < *ρ* < +*∞*). A direction arg*z* = *θ* is called a Borel direction of order *ρ* of *f*(*z*) if, no matter how small the positive number *η* is, for each value *ω*, one has

(44) $$\begin{array}{c} \underset{r\to \infty }{\mathrm{limsup}}\frac{\log n\left(r,\theta ,\eta ,\omega \right)}{\log r}=\rho , \end{array}$$

except for at most two exceptional values *ω*. A direction arg*z* = *θ* is called a Bloch direction of order *ρ* of *f*(*z*) if, for any number *ɛ*  (0 < *ɛ* < *π*/2), any system *a*_*j*_  (*j* = 1,2,…, *q*) of distinct values, and any system *k*_*j*_  (*j* = 1,2,…, *q*) such that *k*_*j*_ is a positive integer or +*∞* and that

(45) $$\begin{array}{c} \sum_{j=1}^{q}\left(1-\frac{1}{k_{j}+1}\right)>2, \end{array}$$

there exists at least one integer *j*  (1 ≤ *j* ≤ *q*) such that

(46) $$\begin{array}{c} \underset{r\to \infty }{\mathrm{limsup}}\frac{\log n^{k_{j})}\left(r,\theta ,ɛ,a_{j}\right)}{\log r}=\rho . \end{array}$$

**Theorem 9**

Let *f*(*z*) be a meromorphic function of order *ρ*  (0 < *ρ* < +*∞*). Then every Borel direction of order *ρ* of *f*(*z*) is a Bloch direction of order *ρ* of *f*(*z*).

In order to prove Theorem 9, we need the following lemma.

**Lemma 10 (Zhang [7])**

Let *f*(*z*) be a meromorphic function of order *ρ*  (0 < *ρ* < +*∞*). Then a direction arg*z* = *θ* is a Borel direction of order *ρ* of *f*(*z*) if and only if it satisfies

(47) $$\begin{array}{c} \underset{r\to \infty }{\mathrm{limsup}}\frac{\log T\left(r,\Omega \left(\theta ,ɛ\right),f\right)}{\log r}=\rho , \end{array}$$

for any *ɛ*  (0 < *ɛ* < *π*/2).

We are now in the position to prove Theorem 9.

**Proof**

Suppose that arg*z* = *θ* is a Borel direction of order *ρ* of *f*(*z*); then, for any *ɛ*  (0 < *ɛ* < *π*/2), we have

(48) $$\begin{array}{c} \underset{r\to \infty }{\mathrm{limsup}}\frac{\log T\left(r,\Omega \left(\theta ,ɛ\right),f\right)}{\log r}=\rho . \end{array}$$

If arg*z* = *θ* is not a Bloch direction of order *ρ* of *f*(*z*), then there exit a system *a*_*j*_  (*j* = 1,2,…, *q*) of distinct values and a system *k*_*j*_  (*j* = 1,2,…, *q*) such that *k*_*j*_ is a positive integer or +*∞* and that

(49) $$\begin{array}{c} \sum_{j=1}^{q}\left(1-\frac{1}{k_{j}+1}\right)>2, \end{array}$$

And, for any integer *j*  (1 ≤ *j* ≤ *q*), we have

(50) $$\begin{array}{c} \underset{r\to \infty }{\mathrm{limsup}}\frac{\log n^{k_{j})}\left(r,\theta ,2ɛ,a_{j}\right)}{\log r}<\rho . \end{array}$$

Hence, we can get

(51) $$\begin{array}{c} \underset{r\to \infty }{\mathrm{limsup}}\frac{\log N^{k_{j})}\left(r,\theta ,2ɛ,a_{j}\right)}{\log r}<\rho , \end{array}$$

for any integer *j*  (1 ≤ *j* ≤ *q*). Therefore, we can find a positive number *τ* < *ρ* such that

(52) $$\begin{array}{c} N^{k_{j})}\left(r,\theta ,2ɛ,a_{j}\right)\leq r^{\tau }. \end{array}$$

By (39), we have

(53) (∑j=1q(1−1kj+1)−2)T(r,Ω(θ,ɛ),f)  ≤∑v=1qkjkj+1Nkj)(r,θ,2ɛ,av)+O(r3ρ/4)  ≤O(rζ),

where *ζ* = max⁡⁡{*τ*, 3*ρ*/4} < *ρ*. Hence,

(54) $$\begin{array}{c} \underset{r\to \infty }{\mathrm{limsup}}\frac{\log T\left(r,\Omega \left(\theta ,ɛ\right),f\right)}{\log r}=\zeta <\rho . \end{array}$$

This contradicts with (48) and Theorem 9 follows.

**Corollary 11**

Let *f*(*z*) be a meromorphic function of order *ρ*  (0 < *ρ* < +*∞*). Then there is a direction arg*z* = *θ* which is a Bloch direction of order *ρ* of *f*(*z*).

Note that Corollary 11 is a corresponding result of Theorem A in angular distribution.

## 5. Pseudo-T Direction of Meromorphic Functions

In 2003, Zheng [1] introduced a new singular direction, called T direction. We call *J* : arg*z* = *θ* the T direction of *f*(*z*), provided that, given any *a* ∈ *ℂ*_*∞*_, possibly with exception of at most two values of *a*, for any positive number *ɛ* < *π*, we have

(55) $$\begin{array}{c} \underset{r\to \infty }{\mathrm{limsup}}\frac{N\left(r,\theta ,ɛ,a\right)}{T\left(r,f\right)}>0. \end{array}$$

For the existence of T direction of meromorphic function *f*(*z*), Guo et al. [10] proved the following Theorem.

**Theorem C**

Let *f*(*z*) be a meromorphic function and satisfy

(56) $$\begin{array}{c} \underset{r\to \infty }{\mathrm{limsup}}\frac{T\left(r,f\right)}{\log^{2}r}=\infty . \end{array}$$

Then *f*(*z*) must have a T direction.

Theorem C was conjectured by Zheng [1]. In [11], the authors study the existence of T direction of *f*(*z*) concerning multiple values. We call *J* : arg*z* = *θ* the T direction of *f*(*z*) concerning multiple values, provided that, given any *a* ∈ *ℂ*_*∞*_, possibly with exception of at most [(2*l* + 2)/*l*] values of *a*, for any positive number *ɛ* < *π*, we have

(57) $$\begin{array}{c} \underset{r\to \infty }{\mathrm{limsup}}\frac{N^{l)}\left(r,\theta ,ɛ,a\right)}{T\left(r,f\right)}>0, \end{array}$$

where [*x*] implies the maximum integer number which does not exceed *x* and *l* is a positive integer.

**Theorem 5 D**

Let *f*(*z*) be a meromorphic function and satisfy (56). Then there at least exists a T direction of *f*(*z*) concerning multiple values.

Note that the T direction of meromorphic function concerning multiple values is a refinement of the ordinary T direction since [(2*l* + 2)/*l*] → 2 as *l* → *∞*. Since Zheng [1] gave the definition of T direction, then there is a considerable number result related this direction, we refer the reader to [12] for finding a careful discussion of this direction.

It is well known that T direction is a concept in angular distribution which corresponds to the Nevanlinna second fundamental theorem in module distribution. It is natural to consider the corresponding result to Theorem B in angular distribution.

**Definition 12**

Let *f*(*z*) be a meromorphic function. A direction arg*z* = *θ* is called a pseudo-T direction of *f*(*z*) if, for any number *ɛ*  (0 < *ɛ* < *π*/2), any system *a*_*j*_  (*j* = 1,2,…, *q*) of distinct values, and any system *k*_*j*_  (*j* = 1,2,…, *q*) such that *k*_*j*_ is a positive integer or +*∞* and that

(58) $$\begin{array}{c} \sum_{j=1}^{q}\left(1-\frac{1}{k_{j}+1}\right)>2, \end{array}$$

there exists at least one integer *j*  (1 ≤ *j* ≤ *q*) such that

(59) $$\begin{array}{c} \underset{r\to \infty }{\mathrm{limsup}}\frac{N^{k_{j})}\left(r,\theta ,ɛ,a_{j}\right)}{T\left(r,f\right)}>0. \end{array}$$

**Theorem 13**

Let *f*(*z*) be a meromorphic function and satisfy (56). Then there at least exists a pseudo-T direction of *f*(*z*).

**Remark 14**

(i) In Theorem C, *q* = 3, *k*_*j*_ = *∞*  (*j* = 1,2, 3), so Theorem C is a special case of Theorem 13.

(ii) If *k*_*j*_ = 1  (*j* = 1,2,…, *q*), then *q* = 5; if *k*_*j*_ = 2  (*j* = 1,2,…, *q*), then *q* = 4; if *k*_*j*_ = *l* ≥ 3  (*j* = 1,2,…, *q*), then *q* = 3. So Theorem D is a special case of Theorem 13.

In order to prove Theorem 13, we need the following lemma.

**Lemma (Li and Gu [13], see also Xuan [14])**

Suppose that Ψ(*r*) is a nonnegative increasing function in (1, *∞*) and satisfies

(60) $$\begin{array}{c} \underset{r\to \infty }{\mathrm{limsup}}\frac{\Psi \left(r\right)}{\log^{2}r}=\infty . \end{array}$$

Then for any set *E* ⊂ (1, *∞*) such that ∫_*E*_(1/*r*log⁡⁡*r*)*dr* < 1/3, one has

(61) $$\begin{array}{c} \underset{r\to \infty ;r\in (1,\infty )-E}{\mathrm{limsup}}\frac{\Psi \left(r\right)}{\log^{2}r}=\infty . \end{array}$$

**Proof**

Firstly, we prove the following statement. Let *m*  (*m* ≥ 4) be a fixed positive integer, *θ*_0_ = 0, *θ*_1_ = 2*π*/*m*,…, *θ*_*m*−1_ = (*m* − 1)2*π*/*m*, *θ*_*m*_ = *θ*_0_. We put Δ(*θ*_*i*_) = {*z* : |arg*z* − *θ*_*i*_| < 2*π*/*m*}, Δ^*o*^(*θ*_*i*_) = {*z* : |arg*z* − *θ*_*i*_| < *π*/*m*},  *i* = 0,1,…, *m* − 1; Δ(*θ*_*m*_) = Δ(*θ*_0_), Δ^*o*^(*θ*_*m*_) = Δ^*o*^(*θ*_0_). Then among these *m* angular domains {Δ(*θ*_*i*_)}, there is at least an angular domain Δ(*θ*_*i*_) such that for any system *a*_*j*_  (*j* = 1,2,…, *q*) of distinct values and any system *k*_*j*_  (*j* = 1,2,…, *q*) such that *k*_*j*_ is a positive integer or +*∞* and that

(62) $$\begin{array}{c} \sum_{j=1}^{q}\left(1-\frac{1}{k_{j}+1}\right)>2, \end{array}$$

there exists at least one integer *j*  (1 ≤ *j* ≤ *q*) such that

(63) $$\begin{array}{c} \underset{r\to \infty }{\mathrm{limsup}}\frac{N^{k_{j})}\left(r,\Delta \left(\theta_{i}\right),a_{j}\right)}{T\left(r,f\right)}>0. \end{array}$$

Otherwise, for any angular domain Δ(*θ*_*i*_)  (1 ≤ *i* ≤ *m*), there is a system *a*_*i*_^*j*^  (*j* = 1,2,…, *q*) of distinct values and a system *k*_*i*_^*j*^  (*j* = 1,2,…, *q*) such that *k*_*i*_^*j*^ is a positive integer or +*∞* and that

(64) $$\begin{array}{c} \sum_{j=1}^{q}\left(1-\frac{1}{k_{i}^{j}+1}\right)>2, \end{array}$$

for any *j*  (1 ≤ *j* ≤ *q*) we have

(65) $$\begin{array}{c} \underset{r\to \infty }{\mathrm{limsup}}\frac{N^{k_{i}^{j})}\left(r,\Delta \left(\theta_{i}\right),a_{i}^{j}\right)}{T\left(r,f\right)}=0. \end{array}$$

Put

(66) $$\begin{array}{c} \sum_{j=1}^{q}\left(1-\frac{1}{k_{j}+1}\right)=\underset{1\leq i\leq m}{\min}\left\{\sum_{j=1}^{q}\left(1-\frac{1}{k_{i}^{j}+1}\right)\right\}>2. \end{array}$$

Applying Theorem 5 to Δ^*o*^(*θ*_*i*+1_), Δ(*θ*_*i*+1_), we have

(67) (∑j=1q(1−1kj+1)−2)T(r,Δo(θi+1),f)  ≤∑j=1qkijkij+1Nkij)(r,Δ(θi+1),ai+1j)   +O(log⁡2r)+χ(r,Δ(θi+1)).

Noting *T*(*r*, *f*) = ∑_*i*=0_^*m*−1^ *T*(*r*, Δ^*o*^(*θ*_*i*+1_), *f*) and adding two sides of the above expression from *i* = 0 to *m* − 1, we can obtain

(68) (∑j=1q(1−1kj+1)−2)T(r,f)  ≤∑i=0m−1∑j=1qkijkij+1Nkj)(r,Δ(θi+1),ai+1j)   +O(log⁡2r)+∑i=0m−1χ(r,Δ(θi+1)).

For any *i*, there exists a *r*_*i*_, the inequality *T*(*r*, Δ^*o*^(*θ*_*i*+1_), *f*) > *e* ^3*m*^ would bold for *r* > *r*_*i*_, while the inequality (22) does not look appropriate here. Put *E*_Δ^*o*^(*θ*_*i*+1_)_ is the set of *r* which consists of a series of intervals and satisfies

(69) $$\begin{array}{c} \int_{E_{\Delta^{o}(\theta_{i+1})}}\frac{1}{r\log r}dr\leq \frac{1}{\log T\left(r,\Delta^{o}\left(\theta_{i+1}\right),f\right)}<\frac{1}{3m}. \end{array}$$

Let *r*_0_ = max⁡⁡{*r*_*i*_, *i* = 1,2,…, *m*}; we have for any *i*, *T*(*r*_0_, Δ^*o*^(*θ*_*i*+1_), *f*) > *e* ^3*m*^; then

(70) $$\begin{array}{c} \int_{\cup_{i=0}^{m-1}E_{\Delta^{o}(\theta_{i+1})}}\frac{1}{r\log r}dr\leq \sum_{i=0}^{m-1}\frac{1}{\log T\left(r,\Delta^{o}\left(\theta_{i+1}\right),f\right)}<\frac{1}{3}. \end{array}$$

Applying Lemma 15, we have

(71) $$\begin{array}{c} \underset{r\to \infty ;r\in (1,\infty )-E}{\mathrm{limsup}}\frac{T\left(r,f\right)}{\log^{2}r}=\infty , \end{array}$$

where *E* = ∪_*i*=0_^*m*−1^ *E*_Δ^*o*^(*θ*_*i*+1_)_. Therefore, there exists a sequence *r*_*n*_′ ∈ (1, *∞*) − *E*, such that

(72) $$\begin{array}{c} \underset{n\to \infty }{\lim}\frac{T\left(r_{n}^{\prime },f\right)}{\log^{2}r_{n}^{\prime }}=\infty . \end{array}$$

It follows from (38), (68), and (72) that

(73) $$\begin{array}{c} \left(\sum_{j=1}^{q}\left(1-\frac{1}{k_{j}+1}\right)-2\right)\leq 0. \end{array}$$

Hence

(74) $$\begin{array}{c} \sum_{j=1}^{q}\left(1-\frac{1}{k_{j}+1}\right)\leq 2. \end{array}$$

This is a contradiction. Hence, for an arbitrary positive integer *m*, there is at least an angular domain Δ(*θ*_*i*_) such that for any system *a*_*j*_  (*j* = 1,2,…, *q*) of distinct values and any system *k*_*j*_  (*j* = 1,2,…, *q*) such that *k*_*j*_ is a positive integer or +*∞* and that

(75) $$\begin{array}{c} \sum_{j=1}^{q}\left(1-\frac{1}{k_{j}+1}\right)>2, \end{array}$$

there exists at least one integer *j*  (1 ≤ *j* ≤ *q*) such that

(76) $$\begin{array}{c} \underset{r\to \infty }{\mathrm{limsup}}\frac{N^{k_{j})}\left(r,\Delta \left(\theta_{i}\right),a_{j}\right)}{T\left(r,f\right)}>0. \end{array}$$

Choosing subsequence of {*θ*_*m*_}, still denote it {*θ*_*m*_}, we assume that *θ*_*m*_ → *θ*_0_. Put *L* : arg*z* = *θ*_0_; then *L* is a pseudo-T direction that is stated in Definition 12.

In fact, for any *ɛ*  (0 < *ɛ* < *π*/2), when *m* is sufficiently large, we have Δ(*θ*_*m*_) ⊂ *Ω*(*θ*_0_, *ɛ*). By (76), we have

(77) $$\begin{array}{c} \underset{r\to \infty }{\mathrm{limsup}}\frac{N^{k_{j})}\left(r,\theta_{0},ɛ,a_{j}\right)}{T\left(r,f\right)}\geq \underset{r\to \infty }{\mathrm{limsup}}\frac{N^{k_{j})}\left(r,\Delta \left(\theta_{m}\right),a_{j}\right)}{T\left(r,f\right)}>0. \end{array}$$

Hence, Theorem 13 holds in this case.
